# *Bacillus* strain BX77: a potential biocontrol agent for use against foodborne pathogens in alfalfa sprouts

**DOI:** 10.3389/fpls.2024.1287184

**Published:** 2024-01-19

**Authors:** Rachel Gollop, Yulia Kroupitski, Ilana Matz, Madhvi Chahar, Moshe Shemesh, Shlomo Sela Saldinger

**Affiliations:** ^1^ Department of Food Science, Institute for Postharvest and Food Science, The Volcani Institute, Agriculture Research Organization, Rishon LeZion, Israel; ^2^ Current address: Department of Bio & Nano Technology, Guru Jambheshwar University of Science & Technology, Hisar, India

**Keywords:** beneficial *Bacillus*, *Salmonella*, *E. coli*, sprouts, seeds, antagonistic activity, biocontrol, disinfection

## Abstract

Despite regulatory and technological measures, edible sprouts are still often involved in foodborne illness and are considered a high-risk food. The present study explored the potential of spore-forming *Bacillus* isolates to mitigate *Salmonella* and *Escherichia coli* contamination of alfalfa sprouts. Food-derived *Bacillus* strains were screened for antagonistic activity against *S. enterica* serovar Typhimurium SL1344 (STm) and enteropathogenic *E. coli* O55:H7. Over 4 days of sprouting, levels of STm and *E. coli* on contaminated seeds increased from 2.0 log CFU/g to 8.0 and 3.9 log CFU/g, respectively. Treatment of the contaminated seeds with the most active *Bacillus* isolate, strain BX77, at 7 log CFU/g seeds resulted in substantial reductions in the levels of STm (5.8 CFU/g) and *E. coli* (3.9 log CFU/g) in the sprouted seeds, compared to the control. Similarly, co-culturing STm and BX77 in sterilized sprout extract at the same ratio resulted in growth inhibition and killed the *Salmonella*. Confocal-microscopy experiments using seeds supplemented with mCherry-tagged *Salmonella* revealed massive colonization of the seed coat and the root tip of 4-day-old sprouted seeds. In contrast, very few *Salmonella* cells were observed in sprouted seeds grown with BX77. Ca-hypochlorite disinfection of seeds contaminated with a relatively high concentration of *Salmonella* (5.0 log CFU/g) or treated with BX77 revealed a mild inhibitory effect. However, disinfection followed by the addition of BX77 had a synergistic effect, with a substantial reduction in *Salmonella* counts (7.8 log CFU/g) as compared to untreated seeds. These results suggest that a combination of chemical and biological treatments warrants further study, toward its potential application as a multi-hurdle strategy to mitigate *Salmonella* contamination of sprouted alfalfa seeds.

## Introduction

Consumption of edible sprouts is increasing globally due to their high nutritional value, in terms of proteins, carbohydrates, minerals, and vitamins (Yang et al., 2013; Mir et al., 2021; Francis et al., 2022). Sprouted seeds are germinated under warm and humid conditions that are also ideal for microbial proliferation (Warriner and Smal, 2014; Galieni et al., 2020; Miyahira and Antunes, 2021). During germination and sprouting, bacteria, including human pathogens, may be present in the seeds and can multiply in and on the newly developing sprouts (Warriner et al., 2003; Kim et al., 2020). Since sprouts are usually consumed raw or lightly cooked, to preserve their nutritional value and organoleptic properties, in cases of contamination, there are no further barriers to eliminate foodborne pathogens before consumption (Yang et al., 2013; Carstens et al., 2019; Mir et al., 2021; Miyahira and Antunes, 2021). Indeed, in recent decades, the contamination of sprouts has become an international food safety concern. Disease outbreaks due to consumption of contaminated sprouts have occurred around the world (Foley et al., 2013; Dechet et al., 2014; Callejon et al., 2015; Carstens et al., 2019; Miyahira and Antunes, 2021; Sarno et al., 2021). The primary foodborne pathogens involved in these outbreaks have been *Salmonella* spp. and enterohemorrhagic *Escherichia coli* strains O157:H7 and O104:H4 (EFSA, 2011; King et al., 2012; Yang et al., 2013; Bayer et al., 2014; Dechet et al., 2014; FDA, 2016; Gensheimer and Gubernot, 2016; Carstens et al., 2019). Thus, the mitigation of sprout contamination by these pathogens is critical for assuring the safety of edible sprouts and preventing sprout-associated outbreaks of disease.

Foodborne illness caused by the consumption of raw sprouts is mainly associated with alfalfa (*Medicago sativa*) sprouts, probably because alfalfa sprouts are among the most widely consumed sprouts (Yang et al., 2013). A common strategy for eliminating the transmission of sprout-borne diseases is the prevention of seed contamination using practices such as Good Agricultural Practices, On-Farm Food Safety Management Practices, Good Manufacturing Practices, and Sanitation Standard Operating Procedures in Primary Production, Postharvest Handling, and Processing (EFSA, 2011; Ding et al., 2013; Miyahira and Antunes, 2021; FDA, 2022). In addition to preventative methods, there are also chemical, physical, and biological methods for eliminating or reducing bacterial populations on seeds (Sikin et al., 2013; Yang et al., 2013; Mir et al., 2021; Miyahira and Antunes, 2021). The National Advisory Committee on Microbiological Criteria for Foods recommends that human pathogens be reduced by 5 logs to enhance sprouted seeds’ safety (NACMCF, 1999). One recommended seed-decontamination treatment is the disinfection of seeds with 20,000 ppm Ca(OCl)_2_ (NACMCF, 1999); however, the efficacy of this and other available treatments is still limited (Ding et al., 2013; Sikin et al., 2013; Yang et al., 2013; Wang et al., 2020; Miyahira and Antunes, 2021). Chemical treatments usually result in an average bacterial reduction of 3.08 log CFU/g (*SD* = 2.03), while high-pressure treatment can yield reductions of up to 5.09 log CFU/g (*SD* = 0.94) (Ding et al., 2013). Still, the commercial application of high-pressure technology in the sprouted-seed industry is limited, mainly due to its high costs (Warriner and Smal, 2014).

Biological treatments to control human pathogens in sprouted seeds are emerging as a sustainable strategy. Those treatments are based on the use of antagonistic microorganisms, also known as protective culture, antimicrobial metabolites, and bacteriophages (Matos et al., 2002; Nandiwada et al., 2004; Matos and Garland, 2005; Fett, 2006; Kocharunchitt et al., 2009; Ye et al., 2009; Ye et al., 2010; Fett et al., 2011; Sikin et al., 2013; Yang et al., 2013; Fong et al., 2017; Shen et al., 2017; Zhang et al., 2019; Kim et al., 2020; Liao et al., 2022). Over the last two decades, only a few studies have evaluated the capacity of bacterial species to control *Salmonella* on sprouted seeds. Among the tested bacteria were the Gram-negative species *Pseudomonas fluorescens* (Matos and Garland, 2005; Fett, 2006; Liao, 2008), *P*. *jenssenii* (Fett, 2006), *Enterobacter asburiae* (Ye et al., 2010), and *Erwinia perscicina* (Kim et al., 2020), and a mixture of the Gram-positive bacteria *Lactobacillus plantarum, Pediococcus acidilactici*, and *Pediococcus pentosaceus* (Rossi and Lathrop, 2019). The biocontrol activity of the tested strains varies, yet some strains do achieve the recommended 5-log CFU reduction (Fett, 2006).

Over the last several decades, members of the spore-forming *Bacillus* group have emerged as biocontrol agents in crop protection (Ji et al., 2013; Fira et al., 2018; Caulier et al., 2019; Chen et al., 2020; Miljakovic et al., 2020; Lahlali et al., 2022). *Bacillus* strains are also used for food preservation and as probiotic foods, and some strains display inhibitory activity against human pathogens (Elshaghabee et al., 2017; Caulier et al., 2019; Elshaghabee and Rokana, 2022). The use of *Bacillus* strains as biocontrol agents takes advantage of their production and secretion of diverse secondary metabolites that possess a broad range of biological functions, their ability to form environmental-resistant endospores, and their ubiquitous distribution in soil, aquatic environments, foods, and the gut microbiota of mammals (Nicholson, 2002; Fira et al., 2018; Caulier et al., 2019; Miljakovic et al., 2020). Specifically, the fact that these strains can be easily cultured for large‐scale fermentation and the robustness of *Bacillus* spores under harsh environmental conditions (Zhang et al., 2020) are important for commercial purposes, considering that the product must be stable during processing and prolonged storage, preferably under ambient temperatures.

Despite the wide use of *Bacillus* species in agriculture (Fira et al., 2018; Caulier et al., 2019; Miljakovic et al., 2020; Lahlali et al., 2022), to date, their use to control human pathogens in sprouts has not attracted much attention (Shen et al., 2017). Recently, we demonstrated the potential use of several spore-forming *Bacillus* strains to inhibit the proliferation of *Salmonella enterica* serovar Typhimurium strain 1344 (STm 1344) during the germination and sprouting of mung bean seeds (Chahar et al., 2023). However, the biocontrol *Bacillus* strains selected in our previous study (Chahar et al., 2023) only slightly inhibited the growth of *Salmonella* in alfalfa sprouts (**Supplementary Figure 1A**). Therefore, in the present study, we focused on isolating *Bacillus* strains that exhibit anti-*Salmonella* activity in sprouted alfalfa seeds. We have identified a potential biocontrol candidate strain, namely, BX77, performed initial taxonomic identification, and analyzed its capacity to inhibit the growth of several *Salmonella* strains during the sprouting of alfalfa seeds.

## Materials and methods

### Bacterial strains and growth conditions

The *Salmonella* strains used in this study included STm SL1344 that is resistant to streptomycin (Gruzdev et al., 2012); STm SL1344 tagged with mCherry (Kroupitski et al., 2015); *S. enterica* serovars Infantis (Lublin et al., 2015), Enteritidis, Virchow, and Hadar (Kroupitski et al., 2009); and *E. coli* O55:H7 (Weiss-Muszkat et al., 2010). Bacterial strains were grown overnight in Difco™ LB, Lennox (BD, Sparks, MD, USA) broth at 37°C with agitation (150 rpm) and then kept as 40% glycerol stocks at -80°C. For the seed-contamination experiments, frozen bacteria were streaked for isolation on LB agar and incubated overnight at 37°C. A single colony from each strain (*Salmonella* serovars Enteritidis, Virchow, and Hadar, and *E. coli*) was inoculated in LB broth or LB broth supplemented with an antibiotic (STm SL1344, 100 µg/mL streptomycin; *Salmonella* serovar Infantis, 30 µg/mL nalidixic acid). Bacteria were grown with shaking (150 rpm) for 18–20 h at 37°C, to generate the inoculum culture for seed contamination.

The *Bacillus* strains used in this study are presented in **Table 1**. *Bacilli* were maintained as spores following growth in LB broth for 18–20 h at 37°C. Briefly, overnight *Bacillus* cultures were washed twice in sterile double-distilled water (SDDW) by centrifugation (2700 g, 10 min) and then re-suspended in 10 ml SDDW. The suspension was heated at 80°C for 20 min to kill vegetative cells and the spores were kept at room temperature until use. Fresh cultures were prepared by first streaking the spores for isolation on LB agar. A single colony was placed into 10 mL LB broth and shaken at 37°C for 18–20 h. The culture was washed in SDDW, as described above, and then re-suspended in 10 mL of sterile water for the seed treatment.

**Table 1:** Inhibition zone of selected *Bacillus* isolates with activity against STm SL1344 and *E. coli*.

Isolate name	STm inhibition zone (mm)	*E. coli* O55:H7 inhibition zone (mm)	Origin of the isolate	Source/ collection
**SP5**	15±2	10±1	Alfalfa sprouts	Sela collection
SP14	12±2	0	Alfalfa sprouts	Sela collection
**SP16**	15±2	0	Alfalfa sprouts	Sela collection
SP18	13±1	0	Alfalfa sprouts	Sela collection
SP19	14±1	0	Alfalfa sprouts	Sela collection
SP20	11±2	0	Alfalfa sprouts	Sela collection
SP22	10±1	0	Alfalfa sprouts	Sela collection
**SP28**	12±2	13±1	Alfalfa sprouts	Sela collection
SP31	10±1	0	Alfalfa sprouts	Sela collection
**SP32**	12±1	12±1	Alfalfa sprouts	Sela collection
SP33	14±2	0	Alfalfa sprouts	Sela collection
SP34	16±4	0	Alfalfa sprouts	Sela collection
SP36	11±1	0	Alfalfa sprouts	Sela collection
SP37	11±1	0	Alfalfa sprouts	Sela collection
**BX77**	16±2	11±1	Dry tea leaves	Sela collection
**P5(S4)**	16±2	15±2	Bovine milk	Shemesh collection
**O5(S2)**	0	14±1	Bovine milk	Shemesh collection
**W2**	12±1	0	Bovine milk	Shemesh collection
**O6(S3)**	14±1	15±2	Bovine milk	Shemesh collection
**YC161**	12±1	12±1	*B. subtilis* Lab strain	Chai, et al, 2011
**MS310**	13±1	13±2	Bovine milk	Shemesh collection

* Isolates' names marked in bold letters were selected for further analysis.

### Seed sprouting

Commercially purchased alfalfa seeds were sprouted in homemade mini-sprouters, as previously described (Chahar et al., 2023). The sprouters containing 1 g of seeds were incubated at 25°C for 4 days in a growth chamber at 92 ± 2% relative humidity.

### Germination rate and determination of sprout growth

Each sprouter contained 1.0 g of seeds (420 ± 9 seeds). We counted the unsprouted seeds among 4-day-old sprouts and used that number to calculate the number of sprouted seeds. The germination rate was determined by dividing the number of the sprouted seeds by the total number of seeds in a single sprouter and multiplying by 100. Each experiment included three sprouters and was repeated three times on different days.

The growth of the sprouts was assessed by measuring their weight and length. The total weight of the sprouts in each of the three sprouters was determined and the average sprout weight was calculated. The measurements were repeated in three separate experiments. Sprout length was measured using a ruler. We measured the lengths of 10 individual sprouts randomly removed from each sprouter (total of 30 sprouts) and then calculated the average length. The measurements were repeated in three independent experiments, including 90 individual sprouts.

### Isolation of spore-forming *Bacillus* strains from sprouts

Four-day-old sprouted seeds (2 g) were collected and bacteria were extracted in 20 mL of SDDW using a stomacher (Bag Mixer, Interscience, France) at maximum power for 2 min. The extract was heated at 80°C for 20 min to select spore-forming bacteria. Ten-fold dilutions of the heated extract were spread on LB agar plates and the plates were then incubated overnight at 37°C. Individual colonies were isolated by streaking on LBGM (LB containing 1% [vol/vol] glycerol and 0.1 mM MnSO_4_) agar plates and then incubating the plates overnight at 37°C. Individual colonies were then streaked again to obtain pure cultures.

### Growth-inhibition assay

The inhibition of *Salmonella* and *E. coli* growth was assessed using the agar-plug diffusion assay (Balouiri et al., 2016). Briefly, spore-forming bacilli were grown overnight on an LBGM agar plate and individual colonies were aseptically excised from the agar and laid over a freshly prepared lawn of *Salmonella* or *E. coli* spread over a nutrient agar plate (NA; BD, Sparks, MD, USA). The plate was incubated at 25°C for 18–20 h and the diameter of the inhibition zone around the *Bacillus* colony was then measured in mm using a ruler. All experiments were repeated four times and the presented data represent the means and standard deviations.

### Antifungal-activity assay

*Aspergillus flavus*, *Fusarium proliferatum*, and *Penicillium expansum* strains were kept at -80°C as glycerol stocks. The fungi were grown on potato dextrose agar (PDA; BD, Franklin Lakes, NJ, USA) at 28°C and maintained for up to 5 days in the dark until use. Conidia were harvested using 5 mL of SDDW and filtered through a 40-µm cell strainer (Biologix, Lenexa, KS, USA) to remove hyphae. Spores were visualized with a BH‐2 series microscope (Olympus, Shinjuku City, Tokyo, Japan) and their concentration was adjusted to 10^6^ CFU/mL in SDDW using a hemacytometer. Twenty-µL drops of each fungal spore suspension were laid on each side of a PDA plate. *Bacillus* suspension containing about 10^7^ CFU/mL was streaked as a line onto the middle of the plate. Control plates contained only fungi. Following drying, the plates were incubated for 7 days at 30°C and the growth inhibition was documented using a digital camera (DXM1200F; Nikon, Tokyo). These experiments were repeated twice.

### Antibacterial activity of *Bacillus* strains in alfalfa sprouts

Alfalfa seeds (1 g) were contaminated with *Salmonella* or *E. coli* at a final concentration of 100 CFU/g (dry seeds) by mixing the seeds with 20 µL of the bacterial suspension containing 5 × 10^3^ CFU/mL. The seeds were dried for 30 min at room temperature and then laid in the sprouter, as previously described (Chahar et al., 2023). In the seed-disinfection experiments (see below), higher concentrations of *Salmonella* (5 × 10^5^ CFU/mL) were also used to contaminate the seeds.

To test for antagonistic activity, a *Bacillus* suspension (10^7^ CFU/mL) was added to the tap water and the sprouters were incubated for 4 days. Each treatment was performed in five sprouters (repeats). The sprouts were weighed and bacteria were extracted by placing the total content of each sprouter in 20 mL of buffer peptone water (BPW; Becton Dickinson) and stomaching for 2 min at maximum speed in a laboratory BagMixer (Interscience BagMixer 400, St. Nom, France). The number of *Salmonella* cells in the sprouted seeds was determined following serial dilutions of the homogenate and plating on XLD (BD, Sparks, MD, USA) agar plates containing streptomycin, as described previously (Chahar et al., 2023).

*E. coli* CFUs were enumerated by plating on HiCrome *E. coli* agar (HiMedia Laboratories, India). Neither *E. coli* nor *Salmonella* was detected in the uncontaminated sprout samples.

### The activity of *Bacillus* against various *Salmonella* serovars in alfalfa sprouts

The antagonistic activity of *Bacillus* was also tested individually against other *Salmonella* serovars (i.e., Infantis, Enteritidis, Virchow, and Hadar). This analysis was performed using both the plug-agar method and sprouted alfalfa seeds. The number of *Salmonella* cells in the sprouted seeds was determined following serial dilutions of the homogenate and plating on Difco™ XLT (BD, Sparks, MD, USA) agar plates; *Salmonella* serovar Infantis was plated on Difco™ XLD agar containing nalidixic acid (30 µg/mL). Each treatment was performed in five sprouters (repeats).

### Disinfection of *Salmonella*-contaminated seeds

Alfalfa seeds (1 g) were inoculated with either 2.0 or 5.0 log CFU/g of STm, as described above. The seeds were dried for 30 min at room temperature and then disinfected by soaking in 5 mL of 20,000 ppm Ca-hypochlorite (Lonza, Arch Chemicals, Inc., Norwalk, CT) for 30 min, with gentle shaking. The seeds were washed five times with 10 mL sterile tap water and laid in the sprouters for 4 days. In some of the sprouters, the water was replaced with a suspension of BX77 (7.0 log CFU/mL). *Salmonella* was counted among 4-day-old BX77-treated sprouted seeds and 4-day-old untreated (control) sprouts, as described above. The BPW extract was incubated in parallel for 24 h at 37°C for *Salmonella* enrichment. If no *Salmonella* colonies appeared on the XLD plates, a sample (0.1 mL) of the enriched culture was spread-plated on XLD agar and examined for typical *Salmonella* colonies.

### Growth curves of *Salmonella* and *Bacillus* during sprouting

The *Salmonella*-inoculated and control seeds were sprouted as described above, with and without the addition of the antagonistic *Bacillus* strain BX77 (7.0 log CFU/mL). *Salmonella* growth in the sprouted seeds was determined once a day for up to 3 days, as described above. Concurrently, the *Bacillus* cell concentration was also determined once daily in *Salmonella*-contaminated and uncontaminated sprouts and untreated sprouted seeds (no *Bacillus* BX77 added). *Bacillus* BX77 colonies were quantified by plating 10-fold dilutions of the sprout extract on LB agar plates and then counting the typical *Bacillus* BX77 colonies, which present a rough morphotype (see **Figure S2**). The lack of distinct *Bacillus* BX77 colonies in the untreated seeds at the tested dilutions confirmed the accuracy of the *Bacillus* enumeration and suggested that the seeds used in this study did not harbor autochthonous *Bacillus* cells at concentrations comparable to those added in the present study. Each treatment was repeated five times.

### Preparation of sprout extract

Three-day-old alfalfa sprouts grown in two sprouters were put into a sterile filter bag (BagFilter^®^, Interscience, France) containing 40 mL SDDW and extracted using a Stomacher instrument for 4 min. The extract was then transferred to a sterile 50-mL conical tube (Greiner, Bio-One GmbH, Germany) and centrifuged at 2700 g for 10 min to remove coarse debris and bacteria. The supernatant was boiled for 10 min to inactivate any residual bacteria and then cooled to room temperature. Finally, the remaining fine particles and residual bacterial debris were removed by filtration through a 0.22-µm PES membrane using a vacuum filter system (Sofra Life Science Research Co., Zhejiang Province, China). The sterility of the extract was confirmed by plating 0.1 mL of the filtrate on an LB plate and then incubating the plate at 25°C for 48 h.

### Enumeration of *Salmonella* and *Bacillus* in the alfalfa extract

*Salmonella* (at a concentration of 10^2^ CFU/mL) was grown in 1 mL of sterile sprout extract in the presence or absence of *Bacillus* BX77 (10^7^ CFU/mL) for up to 3 days at 25°C. Once a day, a sample of 0.1 mL sprout extract was drawn from the tubes and 10-fold dilutions were spread-plated on XLD agar for *Salmonella* enumeration and on LB agar plates for *Bacillus* BX77 counting. Since the *Bacillus* concentrations in the mixed culture were several orders of magnitude higher than those of *Salmonella*, only *Bacillus* colonies were observed on the LB agar in the diluted sprout-extract samples. Furthermore, all colonies had the colony-morphology characteristic of the *Bacillus* BX77 strain. Each experiment was performed twice and repeated three times.

### Fluorescence microscopy

Alfalfa seeds were contaminated with mCherry-tagged STm SL1322 (10^6^ CFU/g), as described above, and sprouted for 4 days in the absence or presence of *Bacillus* BX77 (10^7^ CFU/g). The seeds were analyzed immediately following contamination, using a fluorescent binocular microscope (Nikon SMZ25 Stereo Microscope; Nikon, Japan) and, at 4 days of sprouting, using a confocal laser-scanning microscope (Olympus IX81, Tokyo, Japan). The mCherry-tagged *Salmonella* was visualized using an excitation wavelength of 543 nm and a BA560-600 nm emission filter. The fluorescent images obtained by the microscope were overlaid on the transmitted light images obtained using Nomarski differential interference.

### Bacterial identification by 16S rRNA sequencing

Chromosomal DNA extraction and purification, 16S rRNA gene amplification, and amplicon sequencing were performed as described previously (Chahar et al., 2023). Taxonomic identification was done by sequence analysis using the public EZBioCloud database and tools (Yoon et al., 2017) to determine the similarity to the most closely related species.

### Statistical analysis

Statistical analysis was performed using Instat, version 5.0.4 (GraphPad Software, Inc., La Jolla, CA). One-way analysis of variance (ANOVA) was carried out to identify significant differences within and between groups. The Tukey-Kramer multiple-comparison test was used to compare the means of each group.

## Results

### Isolation of spore-forming *Bacillus* strains with antagonistic activity against STm and *E. coli* O55:H7

**Figure 1:**
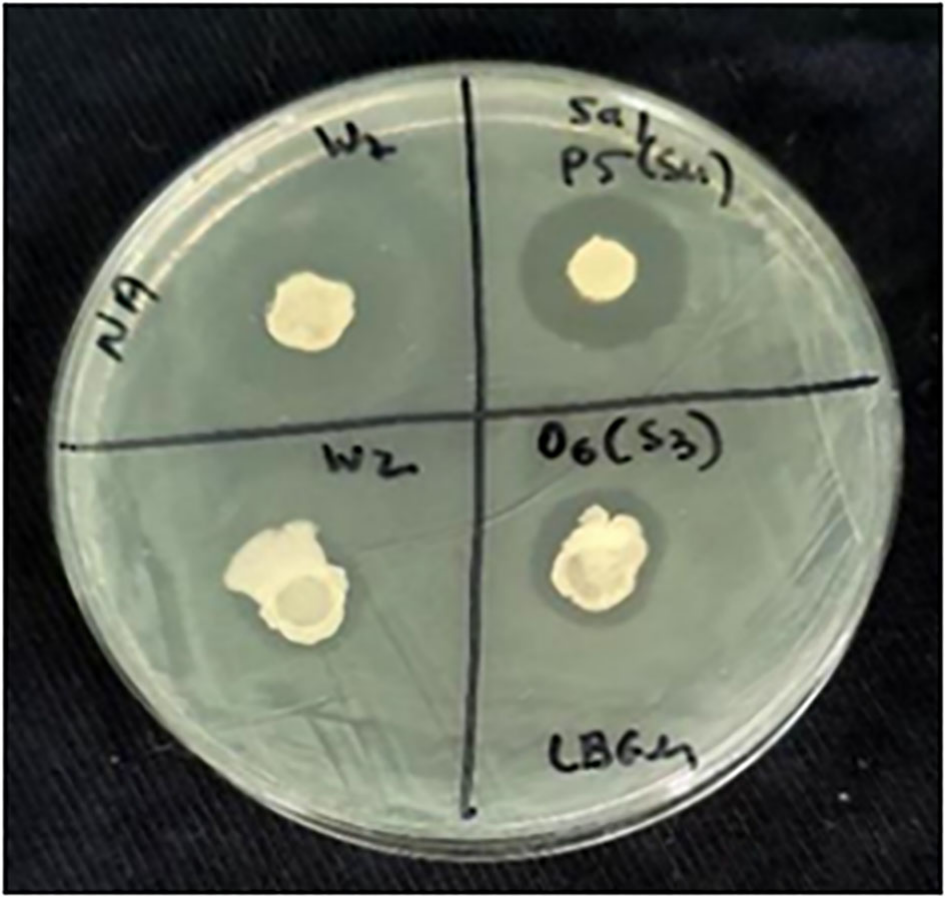
Screening of antagonistic spore-forming bacteria. An example of the screening pipeline showing various inhibition zones of STm SL1344, created by four spore-forming *Bacillus* isolates on a nutrient agar plate.

*Bacillus* strains isolated from alfalfa sprouts and our collections were tested *in vitro* for growth inhibition of STm SL1344 and *E. coli* O55:H7. The observed inhibition zones ranged from zero (no inhibition) to 16 mm, indicating that some *Bacillus* strains secrete antibacterial compounds (**Table 1**; **Figure 1**). Out of 150 *Bacillus* isolates, 21 displayed substantial antibacterial activity. Twenty strains inhibited the growth of *Salmonella* and eight inhibited the growth of both *Salmonella* and *E. coli*. One *Bacillus* isolate (O5-S2) inhibited the growth of *E. coli*, but not *Salmonella*. The *Bacillus* isolates with substantial antibacterial activity were chosen for further analysis.

### Anti-*Salmonella* activity in sprouted alfalfa seeds

**Figure 2:**
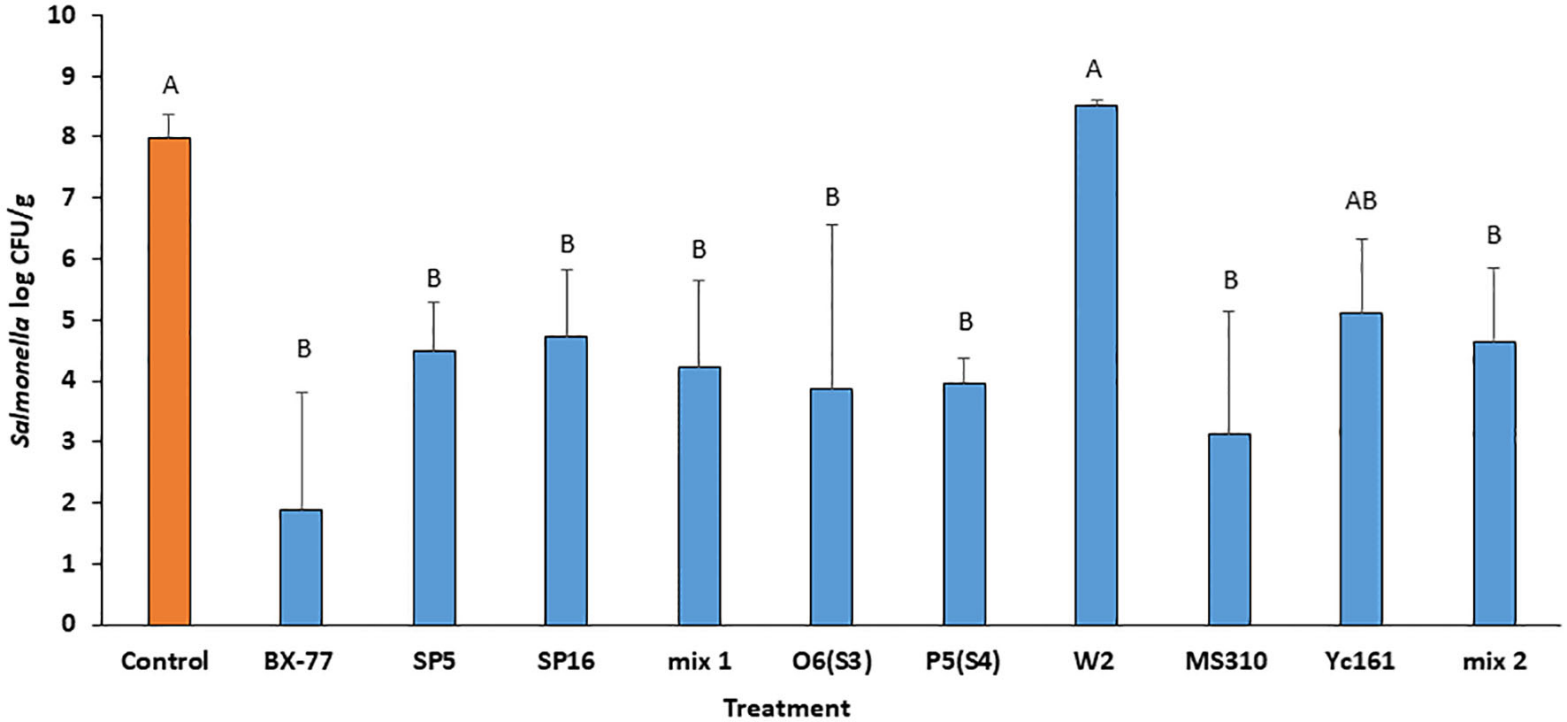
Inhibition of STm SL1344 in 4-day-old alfalfa sprouts by various *Bacillus* isolates and their mixtures. Seeds were contaminated with *Salmonella* (2.0 log CFU/g) and the contaminated seeds were sprouted in the presence or absence (control) of *Bacillus* strains at 7.0 log CFU/mL. Mix 1 was composed of isolates SP5, SP16, and BX77. Mix 2 contained isolates O6(S3), P5(S4), W2, MS310, YC161, and BX77. The data represent the means and standard deviations of five sprouters. Different letters indicate significant differences (*P* ≤ 0.05) between the control and the other treatments.

To test whether the antibacterial activity of the *Bacillus* strains also occurs *in planta*, alfalfa seeds contaminated with *Salmonella* (100 CFU/g) were sprouted for 4 days in the presence or absence of the antagonistic *Bacillus* strains at a concentration of 7.0 CFU/mL. All of the tested strains, except for strain W2R, inhibited the growth of *Salmonella*, compared to a control (**Figure 2**). Among the active strains, BX77 achieved the highest inhibition of *Salmonella* (5.8 log CFU/g), compared to the control. Two mixtures of antagonistic bacteria, Mix 1 and Mix 2, were also tested, to examine a possible additive effect. Mix 1 contained strains SP5, SP16, and BX77, and Mix 2 included strains O6(S3), P5(S4), W2, MS310, YC161, and BX77. Neither of the mixtures enhanced the anti-*Salmonella* activity of BX77 (**Figure 2**). The effectiveness of BX77 against *Salmonella* STm SL1344 was confirmed in three additional experiments, each performed with five sprouters. Treatment of seeds with BX77 inhibited *Salmonella* growth by > 5.0 log CFU/g (**Figure 2**), supporting the notion that this strain could be a potential biocontrol agent.

### Anti-*E. coli* activity in sprouted alfalfa seeds

**Figure 3:**
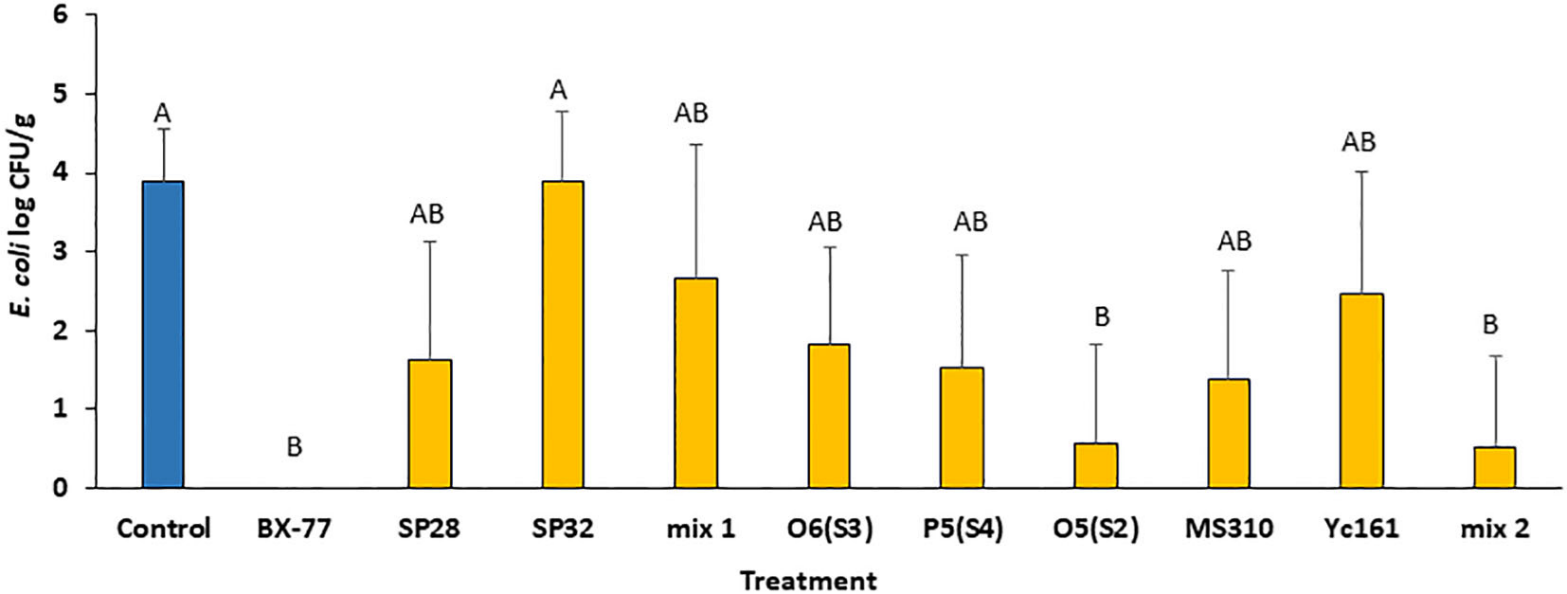
Inhibition of *E. coli* O55:H7 growth in 4-day-old alfalfa sprouts by various *Bacillus* isolates and their mixtures. Seeds were contaminated with *E. coli* (2.0 log CFU/g) and sprouted in the presence or absence (control) of *Bacillus* strains (7.0 log CFU/mL). Mix 1 was composed of isolates SP5, SP16, and BX77. Mix 2 contained isolates O6(S3), P5(S4), W2, MS310, YC161, and BX77. The data represent the means and standard deviations of five sprouters. Different letters indicate significant differences (*P* ≤ 0.05) between the control and the other treatments.

To test whether the antagonistic isolates are also active against pathogens other than *Salmonella*, alfalfa seeds contaminated with *E. coli* O55:H7 were sprouted in the presence or absence of the individual spore-forming strains or their mixtures. The *E. coli* load in 4-day-old alfalfa sprouts reached about 4 log CFU/g after 4 days of sprouting in the absence of *Bacillus* cells (**Figure 3**), compared to 8 log CFU/g in the case of *Salmonella* contamination (**Figure 2**). Supplementation of the contaminated seeds with *Bacillus* cells resulted in the inhibition of *E. coli*. The most antagonistic isolates were BX77 and O5(S2). While BX77 completely inhibited the growth of *E. coli* (3.9 log CFU/g), the addition of O5(S2) resulted in a 3.3 log CFU/g reduction, compared to the control (no *Bacillus* added). As shown for *Salmonella*, the two mixtures of *Bacillus* strains (Mix 1 and Mix 2) were less effective than BX77 alone, with a 1.2 log CFU/g reduction observed for Mix 1 and a 3.4 log CFU/g reduction observed for Mix 2, suggesting the occurrence of bacterial interference.

### Dynamics of *Salmonella* and BX77 populations during alfalfa sprouting

**Figure 4:**
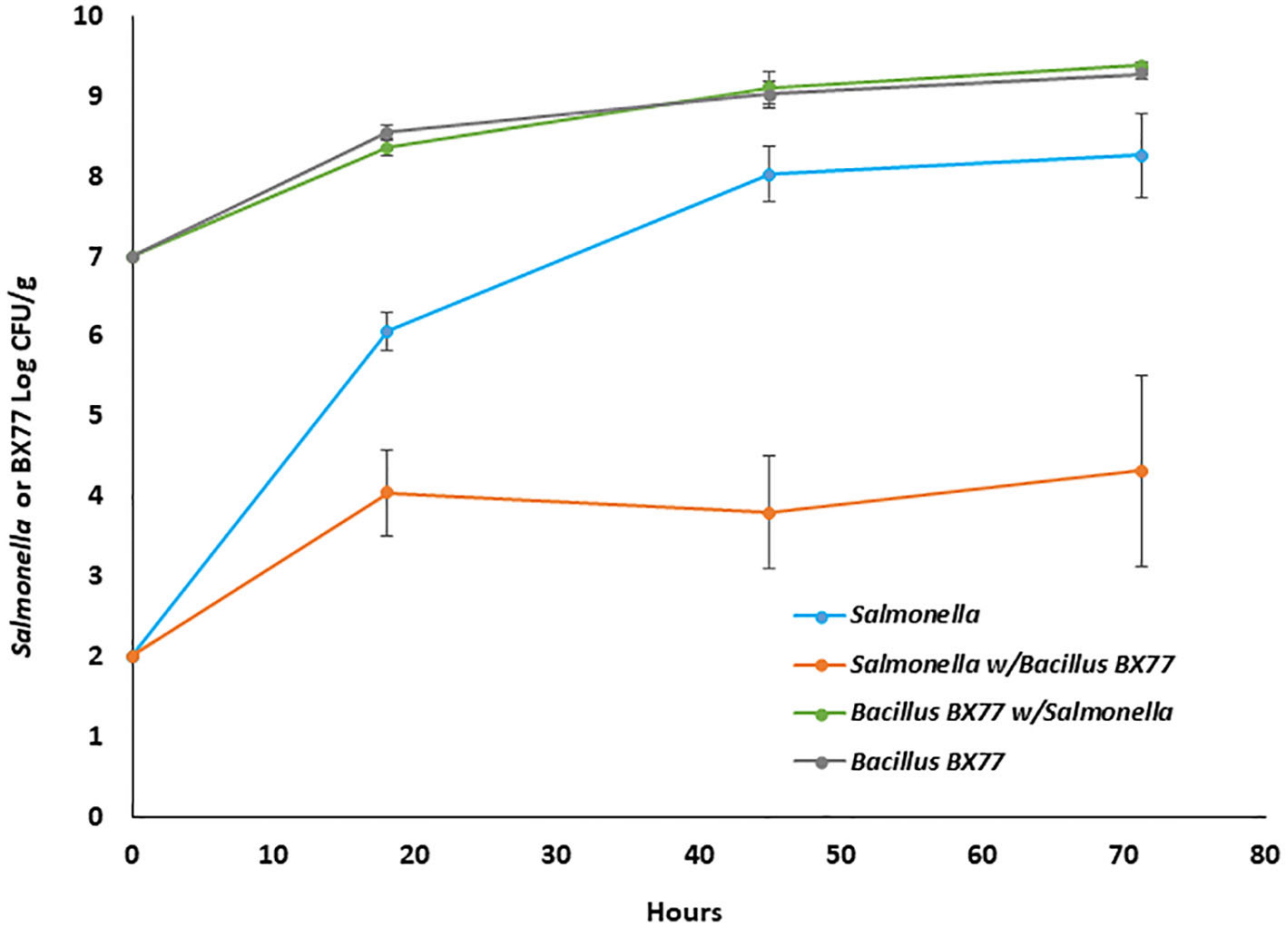
Dynamics of STm and BX77 populations during alfalfa seed sprouting. The data represent the average bacterial population levels at the different time points in a representative experiment with five sprouters. The y-axis refers to CFU/g of either *Salmonella* (blue and orange lines) or *Bacillus* BX77 (green and gray lines).

To study the interactions between BX77 and *Salmonella* during seed sprouting, the growth dynamics of the BX77 and STm populations, alone or in combination, were assessed. Similarly, in separate experiments, seeds were sprouted in the presence of BX77 (no STm added) and the bacterial population was evaluated during seed sprouting (**Figure 4**). During 4 days of sprouting, seeds contaminated with *Salmonella* alone (2.0 log CFU/g) supported pathogen multiplication up to ca. 8 log CFU/g. In contrast, when *Salmonella*-contaminated seeds were sprouted in the presence of BX77 (7.0 log CFU/mL), the rate of *Salmonella* growth was slowed during the first 18 h and the population level remained constant at about 4 log CFU/g for up to 72 h. The BX77 population increased by two orders of magnitude during that time, regardless of the presence of *Salmonella* (**Figure 4**). These findings suggest that the BX77 cells inhibit the growth of STm during the first 18 h and arrest growth from 18 to 72 h.

### Fluorescence microscopy shows that BX77 inhibits the proliferation of *Salmonella* on sprouted seeds

**Figure 5:**
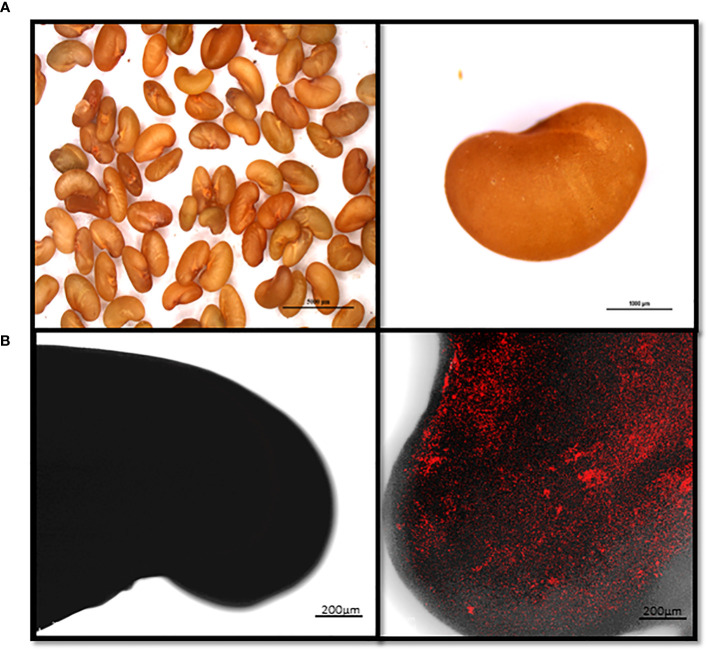
Microscopical images showing attachment of mCherry-tagged *Salmonella* to alfalfa seeds. Seeds were contaminated with mCherry-tagged *Salmonella* (10^6^ CFU/g) and observed under a fluorescent binocular microscope (Nikon SMZ25 Stereo Microscope; Nikon, Japan). Panel **(A)** shows a light image of alfalfa seeds (left) and a magnification of a single seed (right). Panel B shows a representative confocal microscopy image of a non-contaminated seed (left) and a contaminated seed (right). The fluorescent images in panel **(B)** (right) were overlaid with the transmitted light image obtained using Nomarski differential interference. Scale in A left and right panels denote 5000 and 1000 µm, respectively.

**Figure 6:**
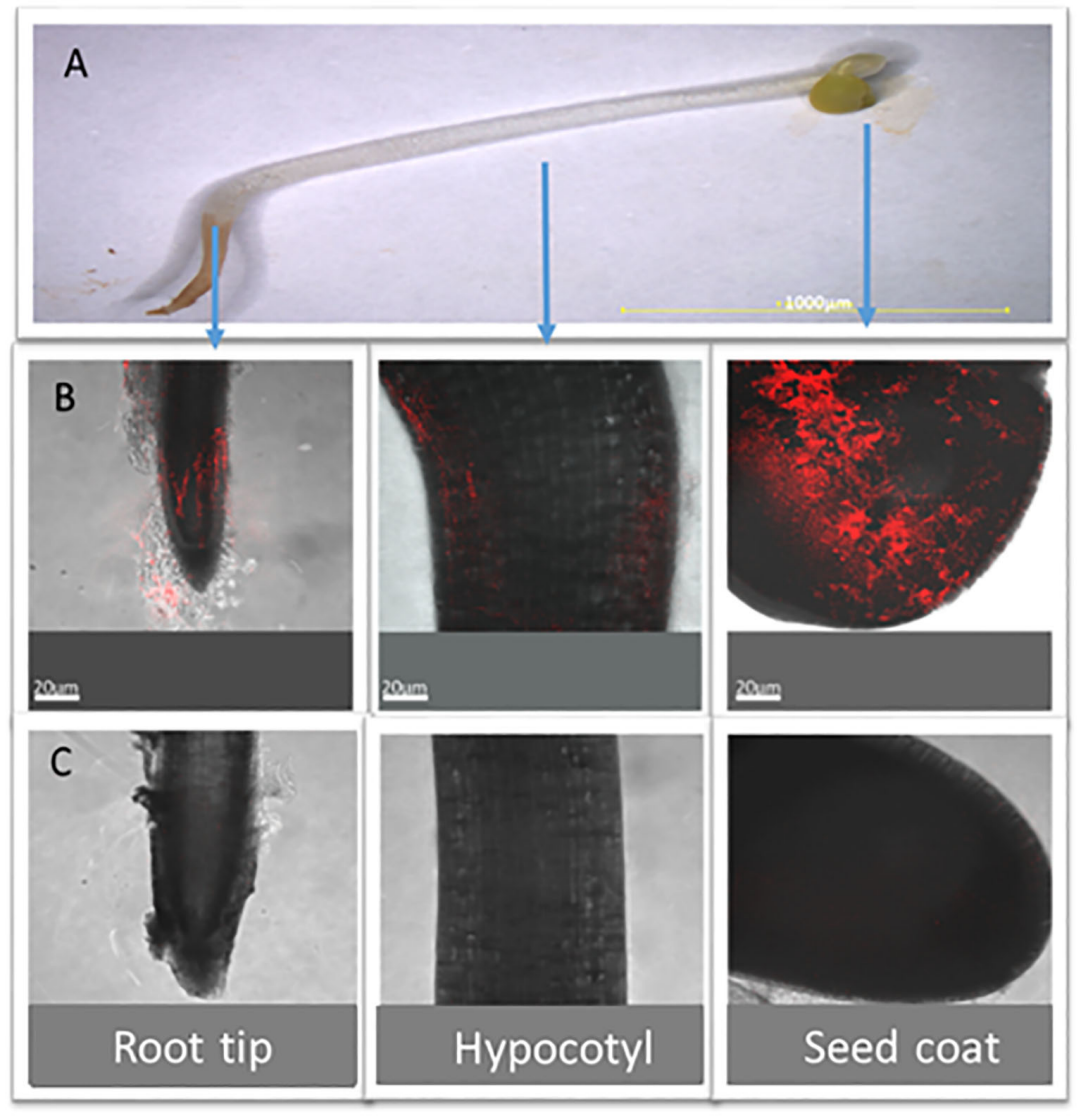
*Salmonella* distribution on different regions of sprouted alfalfa seed in the presence or absence of BX77. Alfalfa seeds were contaminated with mCherry-tagged STm SL1344 (10^6^ CFU/g seeds) in the absence or the presence of BX77 cells (10^7^ CFU/mL). **(A)** shows a light binocular image of a 4-day-old alfalfa sprout (x10). Panel B shows representative confocal microscopy images of mCherry-tagged *Salmonella* (red) on a single alfalfa sprout’s root tip, hypocotyl, and seed coat. **(C)** shows representative confocal microscopy images of the mCherry-tagged *Salmonella* on a seed sprouted in the presence of BX77. The fluorescent images in **(B, C)** were overlaid with the transmitted light image obtained using Nomarski differential interference.

To gain further insight into the antagonistic interactions between the BX77 strain and STm SL1344, the contaminated seeds were examined before sprouting (**Figure 5**) and following 4 days of sprouting (**Figure 6**). Control contaminated seeds (without BX77) were found to be covered with mCherry-tagged *Salmonella*, suggesting that STm SL1344 easily adheres to the entire seed surface. Confocal microscopy of 4-day-sprouted seeds revealed the presence of fluorescent *Salmonella* on various parts of the developing sprout, including the root tip, hypocotyl, and seed coat (**Figure 6B**). In contrast, contaminated seeds sprouted in the presence of the BX77 strain show no or minute amounts of fluorescent *Salmonella* cells (**Figure 6C**), supporting the notion that BX77 interferes with the proliferation of *Salmonella* on sprouted seeds. This finding is consistent with the significant reduction in CFU load presented above (**Figures 2**, **4**).

### BX77 exhibits antagonistic activity against other *Salmonella* serovars

Zone-inhibition assays and *in planta* experiments were used to evaluate the potential activity of BX77 cells against *Salmonella* serovars other than STm SL1344 (**Table 2**). In both of those tests, BX77 cells inhibited the growth of the tested *Salmonella* serovars. In the agar-plug diffusion assay, the zone of inhibition ranged from 8 ± 2 mm in the case of serovars Hadar and Enteritidis to 15 ± 2 mm in the case of serovar Virchow. In the sprout experiments, treatment with BX77 resulted in a reduction of 5.4 to 8.4 log CFU/g of all *Salmonella* serovars (**Table 2**).

**Table 2:** Inhibition of various *S. enterica* serovars by BX77, as determined using zone-of-inhibition and seed-sprouting assays.

*S. enterica* serovar	Inhibition zone diameter (mm)	*Salmonella* log-reduction in sprouts (log CFU/g)*
Typhimurium (SL1344)	14 ± 2	5.61 ± 1.46^A^
Infantis	14 ± 1	6.16 ± 1.97^A^
Enteritidis	8 ± 1	5.41 ± 2.00^A^
Virchow	15 ± 2	8.37 ± 0.00^A^
Hadar	8 ± 2	5.62 ± 1.34^A^

^*^Similar letters indicate no statistical differences (P ≥ 0.05) in the log reduction between STm strain SL1344 and the other Salmonella serovars.

However, there was no direct correlation between the results obtained using the different methods. For example, BX77 displayed relatively low activity against serovars Enteritidis and Hadar on agar plates (8 ± 2 mm). However, it was highly active against these strains in the sprouted-seeds experiments, with reductions of 5.4 and 5.6 log CFU/g, respectively. BX77 exhibited the most potent inhibitory activity *in planta* against serovar Virchow, with a reduction of 8.4 log CFU/g.

### Effects of BX77 on seed germination and sprout development

Since BX77 effectively inhibited the growth of several *Salmonella* serovars in sprouted seeds, it may be considered as a potential biocontrol agent for use against *Salmonella*. To further explore its potential for use as a biocontrol agent, it was important to assess possible adverse effects on seed sprouting, especially since BX77 was present in a relatively high concentration (10^7^ CFU/mL). The seed germination rate and sprout length and weight were examined, to determine whether BX77 has any adverse effects on seed germination or sprout development. The seed germination rate was 81% in the presence of BX77 and 79% in the absence of BX77. Similarly, the addition of BX77 to the sprouted seeds significantly increased (*P* ≤ 0.05) the length of 4-day-old sprouts and did not affect their weight (*P* ≥ 0.05; **Table 3**).

**Table 3:** Effects of BX77 treatment on seed germination and sprout length and weight.

	BX77-treated	Control (No BX77)
Germination^1^ (%)	81 (*N* = 342)^A^	79 (*N* = 331)^A^
Length^2^ (mm)	29.4 ± 7.5 (*N* = 90)^A^	27.1 ± 6.4 (*N* = 90)^B^
Wet weight^3^ (g/sprouter)	3.0 ± 0.5 (*N* = 9)^A^	2.9 ± 0.5 (*N* = 9)^A^

* Different upper-case letters indicate statistical differences (P ≥ 0.05) between treatment and control.

^1^The germination rate was tested in three independent experiments, each performed in triplicate.

^2^We measured the lengths of 30 individual 3-day-old sprouts taken from three sprouters. The measurements were repeated in three independent experiments (90 sprouts total).

^3^The net weight of the sprouts in a single sprouter was measured for each of the three sprouters. The measurements were repeated three times (a total of nine sprouters).

### BX77 exhibits a wide range of antimicrobial activity

**Figure 7:**
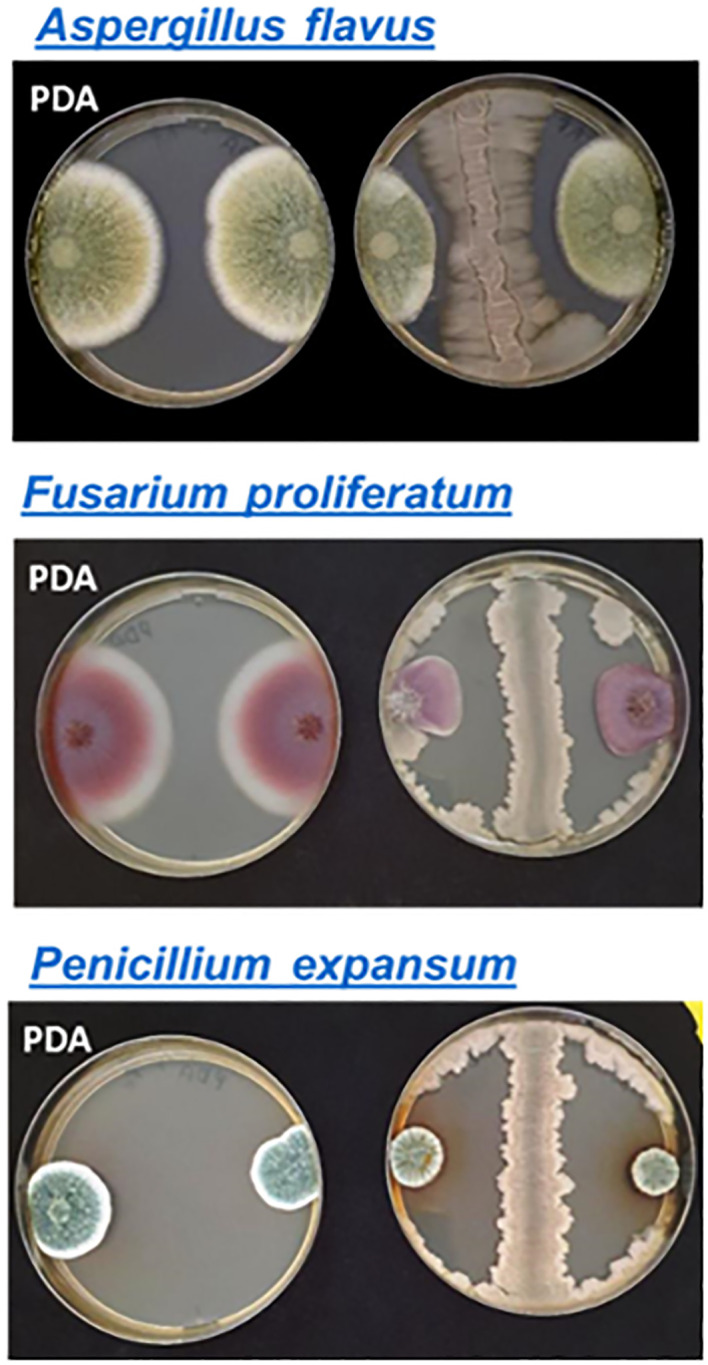
Antifungal activity of BX77. The antifungal activity was demonstrated on PDA using the agar-plate inhibition assay. BX77 appears as a straight line between two fungal colonies on the right side of each panel. Control colonies without BX77 are shown on the left side of each panel.

Strain BX77 was active against several *S. enterica* serovars and *E. coli* O55:H7. Agar-plate assays were employed to gain further knowledge regarding the range of antimicrobial activity of this strain (**Table 4**). BX77 inhibited the growth of several Gram-negative and Gram-positive species, including human and plant pathogens. Yet, it demonstrated no inhibitory activity against the Gram-negative, opportunistic pathogen *Pseudomonas aeruginosa*. In addition to its antibacterial activity, BX77 showed substantial antifungal activity against three plant pathogens: *Aspergillus flavus*, *Fusarium proliferatum*, and *Penicillium expansum* (**Figure 7**). The inhibition of *P. expansum* was associated with the secretion of a brown pigment.

**Table 4:** BX77 inhibits the growth of various bacteria.

Bacterial species	Inhibition diameter (mm)	Comment
*S.* Typhimurium (SL1344)	15 ± 2	Gram-negative, human pathogen
*E. coli* O55:H7	15 ± 2	Gram-negative, human pathogen
*Pseudomonas aeruginosa*	0	Gram-negative, opportunistic human pathogen
*Staphylococcus aureus*	19 ± 2	Gram-negative, human pathogen
*Bacillus cereus*	22 ± 2	Gram-positive, human pathogen
*Listeria innocua*	14 ± 2	Gram-positive, environmental strain
*Pseudomonas fluorescens*	25 ± 2	Gram-negative environmental strain
*Alicyclobacillus acidiphilus*	22 ± 2	Gram-positive, spoilage bacterium
*Pseudomonas syringae*	18 ± 3	Gram-negative, phytopathogen
*Pectobacterium carotovorum*	15 ± 2	Gram-negative, phytopathogen
*Clavibacter michiganensis*	24 ± 3	Gram-positive, phytopathogen

### BX77 enhances Ca-hypochlorite disinfection of contaminated seeds synergistically

**Figure 8:**
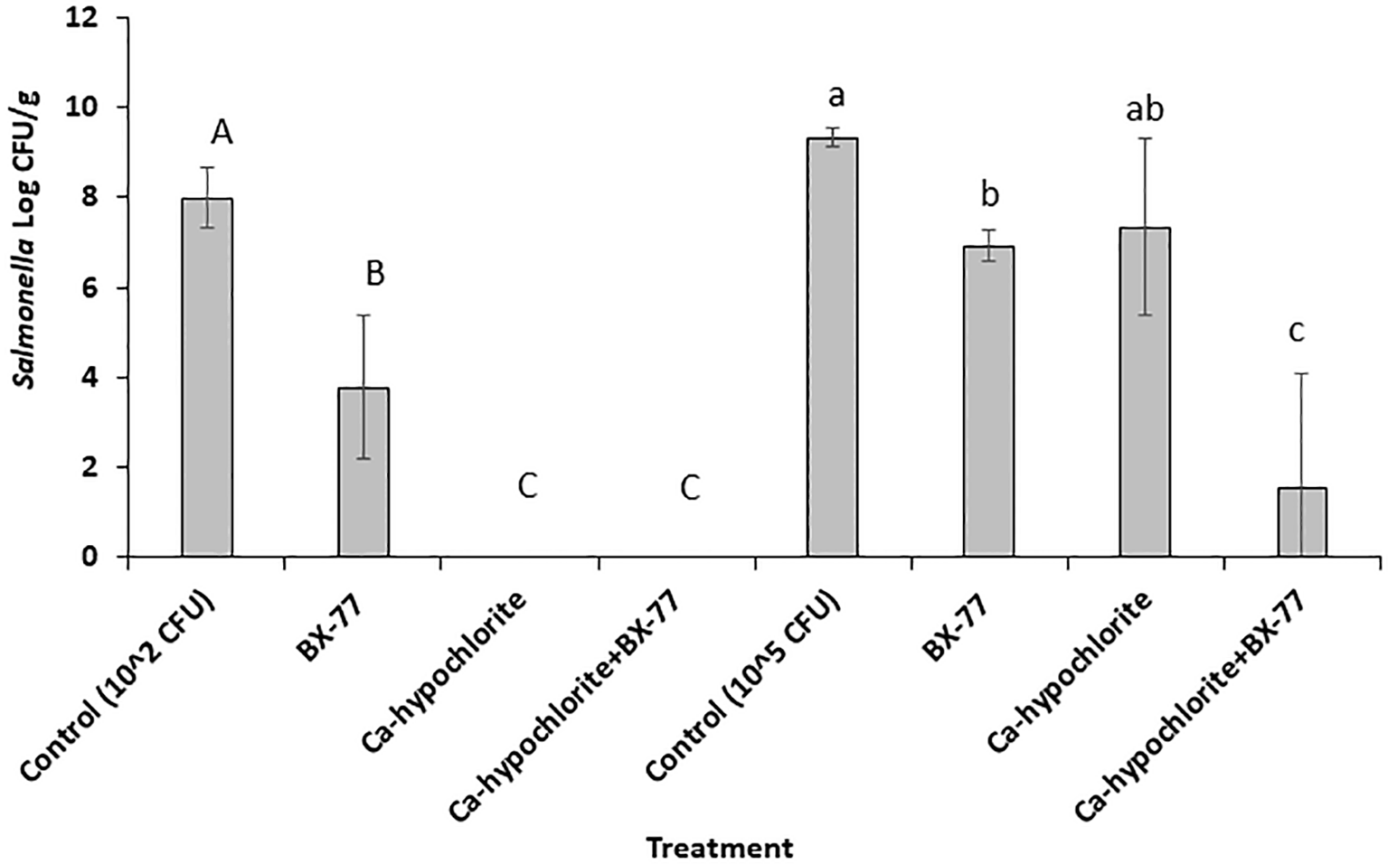
The fate of *Salmonella* in sprouted seeds in the presence or absence of BX77, following seed disinfection with Ca-hypochlorite. The experiments were performed twice, with five repeats for each treatment. The averages and standard deviations are presented. Different letters indicate significant differences (*P* ≤ 0.05) between the control (*Salmonella* alone) and the various treatments.

Alfalfa seeds inoculated with two different *Salmonella* concentrations (2.0 or 5.0 log CFU/g) were disinfected with Ca-hypochlorite, washed, and germinated in the presence or absence of BX77. Non-sanitized *Salmonella-*contaminated seeds, with or without BX77 supplementation, served as controls (**Figure 8**). As shown above, without seed disinfection, *Salmonella* (2.0 log CFU/g) contamination grew to about 8 log CFU/g on the sprouted seeds (considered a low level of contamination). When sprouting occurred in the presence of BX77, *Salmonella* growth was reduced by 4.2 log CFU/g (**Figure 8**). Ca-hypochlorite disinfection of contaminated seeds resulted in the complete eradication of *Salmonella*. Similar results were obtained when the disinfected seeds were sprouted in the presence of BX77.

Inoculation of seeds with a high *Salmonella* concentration (5.0 log CFU/g) yielded comparable results among the non-disinfected seeds. *Salmonella* growth increased from 5 log CFU/g to about 9.3 log CFU/g and sprouting in the presence of the BX77 cells reduced that growth by only 2.4 log CFU/g (**Figure 8**). Disinfection of the contaminated seeds with Ca-hypochlorite had a similar effect (2 log CFU/g reduction). However, when Ca-hypochlorite-treated seeds were sprouted in the presence of BX77, a much greater reduction was observed (7.8 log CFU/g), suggesting a synergistic effect, but not an additive effect.

### Taxonomic identification of the *Bacillus* strain BX77

Taxonomic identification was based on 16S rDNA sequence analysis performed using the EZBioCloud database and tools. The 16S rDNA sequence was deposited in GenBank (accession no. OR743716). The 16 rDNA of the BX77 strain displayed 99.86% similarity to *Bacillus simaensis* strain KCTC 13613 (accession no. AJVF01000043), 99.72% similarity to *B. velezensis* strain CR-502 (accession no. AY603658), and 99.58% similarity to *B. amyloliquefaciens* strain DSM 7 (accession no. FN597644).

### Population dynamics of STm SL1344 and BX77 in alfalfa-sprout extract

**Figure 9:**
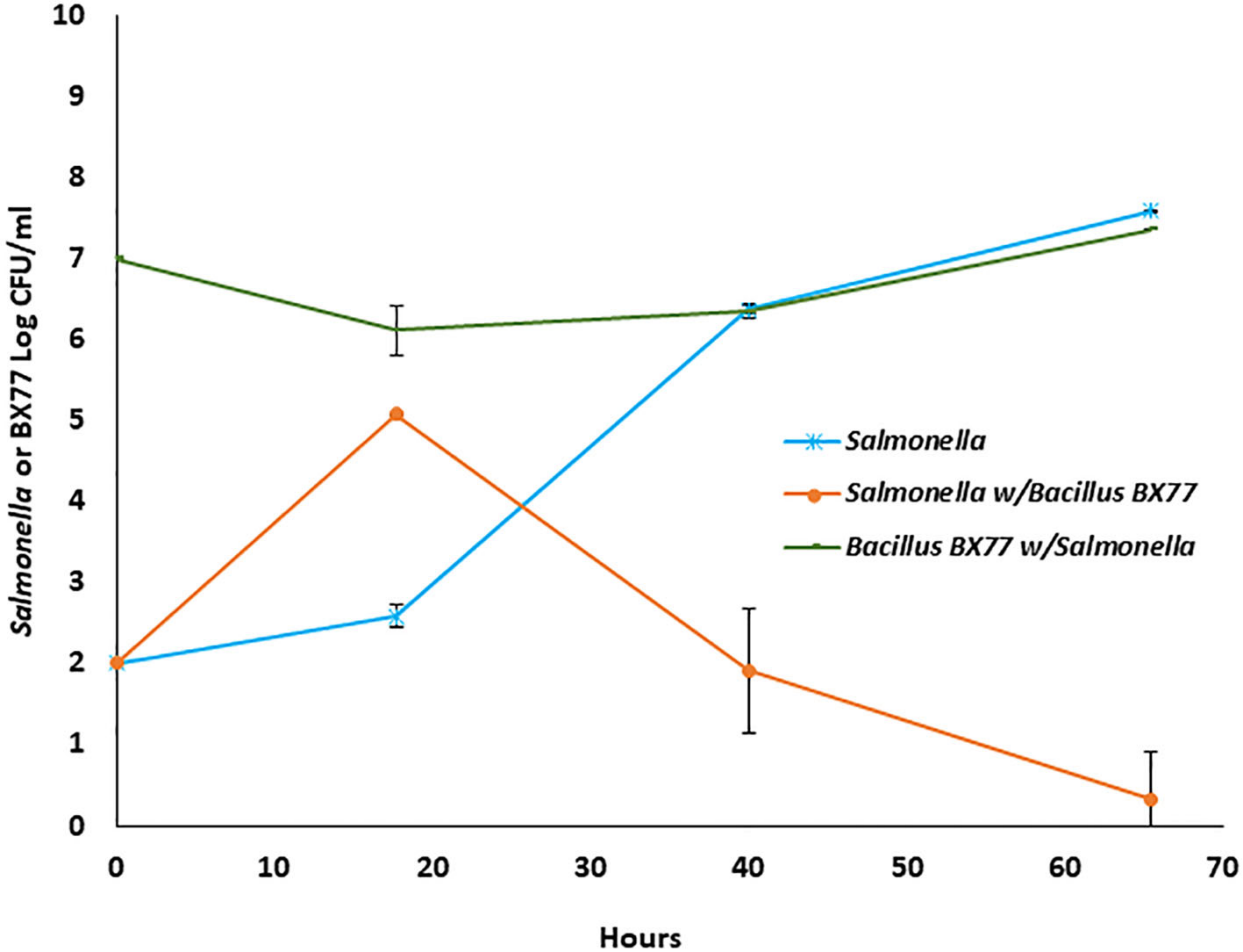
Population dynamics of *Salmonella* STm SL1344 and *Bacillus* BX77 strain in alfalfa sprout extract. The data represent the mean bacterial population at the different time points in two experiments with three repeats. The y-axis refers to CFU/g of either *Salmonella* (blue and orange lines) or *Bacillus* BX77 (green line).

To examine the hypothesis that BX77 may exert at least part of its anti-*Salmonella* phenotype in sprouting seeds by eliciting the plant’s systemic acquired resistance, the effect of BX77 on the growth of STm SL1344 was also tested *ex vivo* by growing the two bacteria as pure or double cultures in sterile sprout extract for 65 h at 25°C. *Salmonella* in a pure culture proliferated from 2.0 log CFU/mL to 7.6 log CFU/mL at 65 h (**Figure 9**). Interestingly, in the presence of BX77, *Salmonella* growth was initially enhanced and reached ca. 5 log CFU/mL at 18 h, but then declined to 1.9 log CFU/mL at 40 h. Further incubation resulted in the *Salmonella* concentration dropping down to 0.3 log CFU/mL at 65 h. The level of BX77 in the mixed culture was slightly reduced initially to 6.1 log CFU/mL at 18 h, but then increased to 7.4 log CFU/mL at 65 h (**Figure 9**).

## Discussion

Controlling human pathogens on plants is a significant challenge for the fresh produce industry. Currently, there are no commercially available means to successfully inactivate these pathogens (Ding et al., 2013; Sikin et al., 2013; Warriner and Smal, 2014; Miyahira and Antunes, 2021). A relatively new approach involves the application of antagonistic microorganisms (Ye et al., 2010; Fett et al., 2011; Sikin et al., 2013; Yang et al., 2013; Uhlig et al., 2021; Chahar et al., 2023).

Relatively few studies have examined the capacity of bacteria to inhibit the growth of human pathogens on sprouting seeds (Matos and Garland, 2005; Fett, 2006; Weiss et al., 2007; Liao, 2008; Ye et al., 2009; Ye et al., 2010; Yang et al., 2013; Kim et al., 2020). In the present study, we sought to explore the potential application of spore-forming *Bacilli* as an alternative treatment to mitigate *Salmonella* contamination of alfalfa sprouts. We identified a potent *Bacillus* strain (BX77) that effectively inhibits the growth of STm SL1344 and *E. coli* O55:H7 in sprouted alfalfa seeds. A partial 16S rDNA sequence showed that this strain is most similar to *B. simaensis* strain PD-A10 isolated from salted crab in Thailand (Sumpavapol et al., 2010). We also observed high degrees of similarity between our sequence and 16S rDNA sequences of strains of *B. velezensis* and *B. amyloliquefaciens*. While precise taxonomic identification of the BX77 strain will require further DNA analysis, based on the current data, this strain seems to belong to the newly proposed taxonomic group ‘operational group *B. amyloliquefaciens*’, which consists of the soilborne *B. amyloliquefaciens* and the plant-associated species, *B. siamensis* and *B. velezensis* (Fan et al., 2017). Members of this taxonomic group include important plant biocontrol and growth-promoting strains (Fan et al., 2017; Fan et al., 2018; Rabbee and Baek, 2020; Ngalimat et al., 2021; You et al., 2021; Wang et al., 2022; Gorai et al., 2023; He et al., 2023; Sharma et al., 2023), some of which are already in commercial use (Fan et al., 2017; Fan et al., 2018).

The addition of BX77 to seeds inhibited the growth of *Salmonella* (> 5 log) and *E. coli* O55:H7 (~ 4 log CFU/g) over 4 days of sprouting. It should be noted that the tested *E. coli* strain grew to a lesser extent in the sprouting seeds than *Salmonella* did and the number of *E. coli* CFU/g in the treated sprouts was below the detection level. Our results are in line with previous reports, which showed inhibition of *S. enterica* on sprouted seeds by non-spore-forming bacteria. For example, Vitt et al. (2023) recently reported that plant-associated bacteria, including members of the *Pseudomona* and *Pantoea* genera, reduced *Salmonella* growth in alfalfa sprouts from ca. 10^8^ to ca. 10^5^ CFU/g (Vitt et al., 2023). Liu and co-workers found that *P. fluorescenes* A506, a known biocontrol agent, inhibited the attachment of *S. enterica* and enterohemorrhagic *E. coli* strains to alfalfa seeds, as well as other vegetable seeds. However, these strains had only mild inhibitory effects on pathogen growth *in vitro* (Liu et al., 2021).

Matos and Garland (2005) tested the antimicrobial activity of *P. fluorescens* 2-79 strain against a cocktail of four *Salmonella* serovars (Anatum, Infantis, Newport, and Stanley) on alfalfa sprouts (Matos and Garland, 2005). The *Pseudomonas* strain strongly inhibited *Salmonella* in 1- to 3-day-old sprouts (> 4 log CFU/g), but that effect faded over time (1.81 log CFU/g on Day 7). Fett (2006) also described strong inhibitory activity of *P. fluorescens* 2-79 against a cocktail of *S*. *enterica* serovars on sprouted alfalfa seeds, with an average reduction of 5 log CFU/g in 6-day-old sprouts, confirming the antagonistic activity of this strain in sprouting seeds (Fett, 2006).

A year later, Weiss and co-workers (2007) selected another *Pseudomonas* species, *P*. *jenssenii* LTH5930, from mung bean sprouts and reported that this strain was able to reduce the growth of *S*. *enterica* serovar Senftenberg in sprouted mung bean seeds by > 3 log CFU and >2 log CFU/g, compared to untreated seeds, after 24 and 48 h, respectively (Weiss et al., 2007). In another series of experiments, mung bean seeds were treated with *P*. *jenssenii* on Day 0 (8 log CFU/g) and Day 1, and then contaminated with *Salmonella* serovar Senftenberg (0.1–1.0 log CFU/g). After 2 days of germination, *Salmonella* was not detected in the *P*. *jenssenii*-treated seeds; in contrast, *Salmonella* grew up to 10^4^ CFU/g in the untreated seeds. After 7 days, *P*. *jenssenii*-treated sprouts harbored <10 CFU/g *Salmonella*, compared to 10^8^ CFU/g in the untreated control group (Weiss et al., 2007). In another study, Ye et al. (2010) tested the efficacy of *Enterobacter asburiae* for controlling *Salmonella* on mung bean sprouts. They observed a slight inhibitory effect (1.16 ± 2.14 log CFU/g); however, a combination of the bacterium and bacteriophages substantially inhibited the growth of *Salmonella* in both mung bean and alfalfa sprouts (> 6 log CFU/g) (Ye et al., 2010).

Kim et al. (2020) used a non-antagonistic *Erwinia persicina* strain (EUS78) to control *Salmonella* in alfalfa sprouts. Those authors showed changes in the population density of *Salmonella enterica* on sprouted alfalfa from 4.4 log CFU/g to 0.5 log CFU/g after 72 h of sprouting in the presence of *Erwinia persicina* strain EUS78 and significant inhibition (> 3 log CFU/g) of the pathogen after 6 days of sprouting. Since that *Erwinia* strain showed no antibacterial activity against *S. enterica*, the authors concluded that the observed biocontrol activity was mediated by competitive exclusion (Kim et al., 2020).

Gram-positive, non-spore-forming bacteria have also been examined for their potential use as biocontrol agents against human pathogens in sprouts. Rossi and Lathrop (2019) evaluated the efficacy of a mixture of *Lactobacillus plantarum*, *Pediococcus acidilactici*, and *Pediococcus pentosaceus* against *Listeria monocytogenes* and *Salmonella* on alfalfa sprouts. Those researchers found only a slight reduction of *Salmonella* compared to the control (1.0 log CFU) at 5 days of sprouting (Rossi and Lathrop, 2019). More recent studies have found that *B. subtilis* ATCC 6051 and *B. mojavensis* RRC 101 compete with *Salmonella* and *E. coli* for attachment to alfalfa seeds (Liu et al., 2021). Even more recently, Vitt et al. (2023) reported that a Gram-positive strain of the genus *Priestia* isolated from alfalfa is capable of inhibiting the growth of *Salmonella* in sprouted alfalfa seeds (Vitt et al., 2023).

Unlike non-spore-forming bacteria, spore-forming strains can make very good biocontrol agents, since their spores are very robust (Nicholson, 2002; Zhang et al., 2020) and can be stored for long periods at room temperature. This feature reduces the costs associated with cold storage or freeze-drying required for vegetative bacteria. Until now, only two studies have evaluated the biocontrol potential of spore-forming bacilli against human pathogens in sprouting seeds. The first study reported the inhibition of enterohaemorrhagic *E. coli* cocktail internalization in sprouted mung bean seeds by *B. subtilis* LCA1 strain and found a 2.0 log CFU/g reduction on Day 5 of sprouting. A smaller reduction (1.1–1.4 log CFU/g) in internalization was achieved using cocktails of *S. enterica* strains (Shen et al., 2017).

More recently, we have shown that several mung bean-derived *Bacillus* strains can inhibit the growth of several *S*. *enterica* serovars on sprouted mung bean seeds. The most active strains inhibited the growth of *S*. *enterica* serovars Typhimurium, Hadar, Virchow, and Enteritidis by > 5.0 log CFU/g, as compared to untreated sprouts. In addition, a mild inhibitory effect (2.7 ± 0.3 and 2.6 ± 1.5 log CFU/g reduction) has been observed against *S*. *enterica* serovar Infantis (Chahar et al., 2023). Notably, the mung bean-derived strains (BX-5 and BX-6) inhibited *Salmonella* growth in sprouted alfalfa seeds only slightly (**Supplementary Figure 1A**). Unlike the case involving alfalfa sprouts, BX77 only slightly inhibited the growth of *Salmonella* in mung bean sprouts (**Supplementary Figure 1B**). This finding supports the notion that the observed antagonistic activity is species- or strain-specific and that different types of host plants may require the development of specifically adapted biocontrol strains. It is possible that the sprouts’ environment may modulate the ability or the level of activity of *Bacillus* strains by triggering the production of specific secondary metabolites that exhibit anti-*Salmonella* activity. Alternatively, diverse *Bacillus* strains may differentially trigger the host immune system to resist *Salmonella* colonization.

The biocontrol activity of *Bacillus* against phytopathogens may be mediated by antibiosis, competition, and/or the induction of systemic resistance response (Santoyo et al., 2012; Chowdhury et al., 2015; Fan et al., 2017; Fira et al., 2018). In the case of *B. amyloliquefaciens* FZB42 and related strains of the operational group *B. amyloliquefaciens*, stimulation of the plants’ systemic resistance by secondary metabolites plays a key role in the mechanism of biocontrol action (Chowdhury et al., 2015). Therefore, it is possible that the sprouts’ systemic resistance is also involved in the activity of BX77 against human pathogens.

To begin to understand the mechanism by which BX77 inhibits the growth of *Salmonella* in alfalfa sprouts, we examined the interactions between BX77 and STm SL1344 *ex vivo* using a sterile alfalfa-sprout extract as a growth medium. *Salmonella* grew well in the extract, reaching 7.6 log CFU/mL. However, in a dual culture, *Salmonella* growth was initially enhanced in the presence of BX77 strain up to 18 h and reached about 5 log CFU/mL. Yet, subsequent incubation resulted in the substantial killing of the pathogen and the viable count declined to 0.3 log CFU/mL at the end of the experiment (65 h), corresponding to a >7 log CFU/g reduction. Since the counts of BX77 in the presence of *Salmonella* remained almost constant throughout the incubation period, these findings suggest that following an adaptation period (18 h), BX77 likely produces and secretes secondary metabolites with anti-*Salmonella* properties. The two strains are unlikely to compete for nutrients, as the net growth of BX77 in the dual culture was negligible. However, unlike in the extract environment, nutrients are not freely available in intact sprouts. It is noteworthy that the BX77 strain only inhibited the growth of *Salmonella in planta*, while massive killing was demonstrated in the sprout extract. It is conceivable that the relatively mild activity against *Salmonella* in alfalfa is related to interfering microbial populations and plant-derived metabolites in the intact-sprout environment. Future studies should elucidate the mechanism by which BX77 inhibits the growth of *Salmonella in planta*.

BX77 displays broad antimicrobial activity against multiple bacteria and fungi, a characteristic likely associated with the plethora of antimicrobial molecules produced and secreted by members of the ‘operational group *B. amyloliquefaciens*’ (Fan et al., 2017; Fan et al., 2018; Kiesewalter et al., 2021; Huang et al., 2022; Gorai et al., 2023; Iqbal et al., 2023). Substantial antibacterial activity was shown against various strains of virulent *S. enterica* serovars (Enteritidis, Hadar, Virchow, and Infantis), both *in vitro* and *in planta*. BX77 also inhibited several phytopathogens (*Pseudomonas syringae*, *P. fluorescence*, *Pectobacterium carotovorum*, and *Clavibacter michiganensis*) and human pathogens (*E. coli* O55:H7, *S. aureus*, and *B. cereus*), as well as the spoilage bacterium *Alicyclobacillus acidiphilus*. In addition, BX77 displayed antifungal activity against three plant pathogens: *Aspergillus flavus, Fusarium proliferatum*, and *Penicillium expansum*.

*Bacillus* strains are well known for their vast repertoire of antimicrobial molecules, including cyclic lipopeptides, such as surfactins and ribosomal and non-ribosomal peptides, and numerous volatile organic compounds, whose nature and concentrations vary between strains (Caulier et al., 2019; Rabbee and Baek, 2020; Kiesewalter et al., 2021). While some of these molecules may be associated with the antimicrobial activity of BX77, further studies are required to identify the nature of the molecules associated with the observed antimicrobial activity.

Seeds for sprout production usually undergo a decontamination treatment prior to germination, to eliminate human pathogens and enhance the safety of the sprouted seeds (Ding et al., 2013; Sikin et al., 2013; Yang et al., 2013; Mir et al., 2021). Disinfection with Ca(OCl)_2_ is presently the most common decontamination technology. However, such treatment is inefficient for the elimination of human pathogens in alfalfa seeds (Beuchat et al., 2001; Montville and Schaffner, 2004; EFSA, 2011; Ding et al., 2013; Sikin et al., 2013; Yang et al., 2013; Wang and Kniel, 2014). The efficacy of seed-decontamination procedures is usually much lower (Ding et al., 2013; Wang et al., 2020; Miyahira and Antunes, 2021) than the recommended 5-log reduction level (NACMCF, 1999). To test whether combined chemical and biological treatments could provide more effectively eradicate *Salmonella* from sprouted alfalfa seeds, we used alfalfa seeds contaminated with low (2 log) or high (5 log CFU/g) levels of the pathogen. Ca-hypochlorite effectively controlled low-level contamination, eradicating the pathogen in those situations. In contrast, in the case of the high level of *Salmonella* contamination, the decontamination treatment resulted in only about a 2 log CFU reduction, similar to previous reports (Beuchat et al., 2001; Liao, 2008). However, adding BX77 to the chemically decontaminated seeds led to a further *Salmonella* reduction, resulting in 7.8 log CFU/g reduction in 4-day-old sprouts, compared to untreated sprouts. These findings suggest that, in the case of high contamination levels, neither chemical decontamination nor a biocontrol treatment alone may achieve the recommended 5-log reduction target (NACMCF, 1999). However, the combined treatment may be a suitable alternative, possibly due to a synergistic effect between the chemical and biological treatments, making it a potential practical treatment in the sprout industry. A combined chemical and biological treatment was previously tested (Liao, 2008) to overcome the limitation of chlorine disinfection. However, in that work, the combination of Ca-hypochlorite and *P. fluorescence* 2-79 treatments resulted in a 2 log CFU/g reduction of *Salmonella* compared to the non-sanitized control, which is lower than the CFU reduction obtained in the present study. Reducing the seed-associated microflora may enhance the multiplication of human pathogens introduced during germination and sprouting (Liao, 2008). Thus, adding antagonistic bacteria to the sanitized seeds may also lower post-treatment contamination risk during sprouting and, possibly, postharvest contamination as well. Further studies are needed to examine this possibility.

To further examine the potential use of BX77 as a biocontrol agent, it was important to assess possible adverse effects on seed sprouting. Our results showed that seed germination rates were similar in the presence or absence of BX77. Similarly, the addition of BX77 to sprouted seeds significantly increased sprout length and did not affect the weight of 4-day-old sprouts.

Confocal-microscopy analysis of *Salmonella* on alfalfa seeds revealed that the pathogen adheres to the seed coat and spreads to the hypocotyl and the root tip during germination and sprouting. Similar findings have also been reported for other *Salmonella* serotypes’ colonization of alfalfa sprouts (Charkowski et al., 2002). These findings indicate that *Salmonella* migrates in the growing sprout and may colonize various organs. The seed coat harbors the most significant numbers of the pathogen, apparently because it is the site of contamination. Treatment with BX77 considerably reduced *Salmonella* colonization of the various sprout organs, which is congruent with our enumeration of viable CFU. Similar behavior of *Salmonella* in untreated or *Bacillus*-treated sprouted mung bean seeds was shown previously (Chahar et al., 2023).

Finally, the data presented in this manuscript support the idea that *Bacillus* strains, in addition to their current use in plant protection, have the potential to be used as biocontrol agents against human pathogens on plants. We previously studied the biocontrol of *Salmonella* in mung bean sprouts using several spore-forming *Bacillus* strains (Chahar et al., 2023). Notably, those strains are not very effective against *Salmonella* in alfalfa sprouts (**Supplementary Figure 1A**) and BX77 exhibits low anti-*Salmonella* activity in sprouted mung bean seeds (**Supplementary Figure 1B**). These findings indicate that a “one (biocontrol) strain fits all” approach may not be suitable for controlling human pathogens in plants. It seems that specific *Bacillus* strains may be required for controlling human pathogens in different types of sprouts. Since BX77 exerts greater anti-*Salmonella* activity *in vitro*, it is likely that species-specific host-plant factors regulate the production and secretion of antimicrobial molecules in the intact sprouts. The mechanisms involved in *Bacillus*–plant interactions warrant further research. Additional studies will be required to target the biocontrol agent to a specific host plant. The use of a cocktail of *Bacillus* strains could be a viable strategy for the development of a commercial biocontrol agent.

Our study paves the way toward the development of BX77 as a biocontrol agent for use in alfalfa, especially in combination with a seed-decontamination step to minimize the health risk associated with *Salmonella* contamination. Further studies will be required to assess the activity of BX77 against other pathogenic strains of *S. enterica* and *E. coli* and other human pathogens in the sprouting-seed environment. Lastly, the safety of BX77 should be determined, as a critical step toward the commercialization of this strain as a biocontrol product.

## Conclusions

Antagonistic *Bacillus* strains may be employed as sustainable biocontrol agents to mitigate *Salmonella* contamination of sprouted alfalfa seeds. Distinct *Bacillus* biocontrol strains should be selected for use with different plant hosts. A multi-hurdle approach that combines a chemical decontamination treatment with a biological treatment could be an effective way to reduce *Salmonella* contamination in alfalfa sprouts. That said, the combined treatment examined in this work has the potential to serve as a practical technology to enhance the safety of sprouted seeds.

## Data availability statement

The datasets presented in this study can be found in online repositories. The names of the repository/repositories and accession number(s) can be found below: GenBank, OR743716.

## Author contributions

RG: Investigation, Writing – review & editing, Formal Analysis, Validation, Writing – original draft. YK: Formal Analysis, Investigation, Writing – review & editing. IM: Investigation. MC: Writing – original draft. MS: Conceptualization, Funding acquisition, Writing – review & editing, Supervision. SSS: Conceptualization, Funding acquisition, Writing – review & editing, Investigation, Supervision.

## Supplementary material

Supplementary Figure 1 Reduction of *Salmonella* in alfalfa and mung bean sprouts by various *Bacillus* isolates. Seeds were contaminated with *Salmonella* (2.0 log CFU/g) and sprouted for 4 days in the presence or absence (control) of *Bacillus* strains at a concentration of 7.0 log CFU/mL. **(A)** Sprouted alfalfa seeds. **(B)** Sprouted mung bean seeds. *Bacillus* strains BX5 and BX6 were isolated from mung bean sprouts and described previously (Chahar et al., 2023). The data represent the means and standard deviations of two independent experiments, each with three replicates. Different letters indicate significant differences (*P* ≤ 0.05) between the tested strains.

Supplementary Figure 2 Enumeration of *Bacillus* BX77 colonies on LB agar plates following extraction from treated sprouted seeds. No *Bacillus* BX77-like colonies were recovered from untreated sprouted-seed extract at similar dilutions.
