# Supplementary figures and images for: Bacillus strain BX77: a potential biocontrol agent for use against foodborne pathogens in alfalfa sprouts
Source: Front Plant Sci. 2024 Jan 19;15:1287184. doi: 10.3389/fpls.2024.1287184 (PMC10834763; doi:10.3389/fpls.2024.1287184)

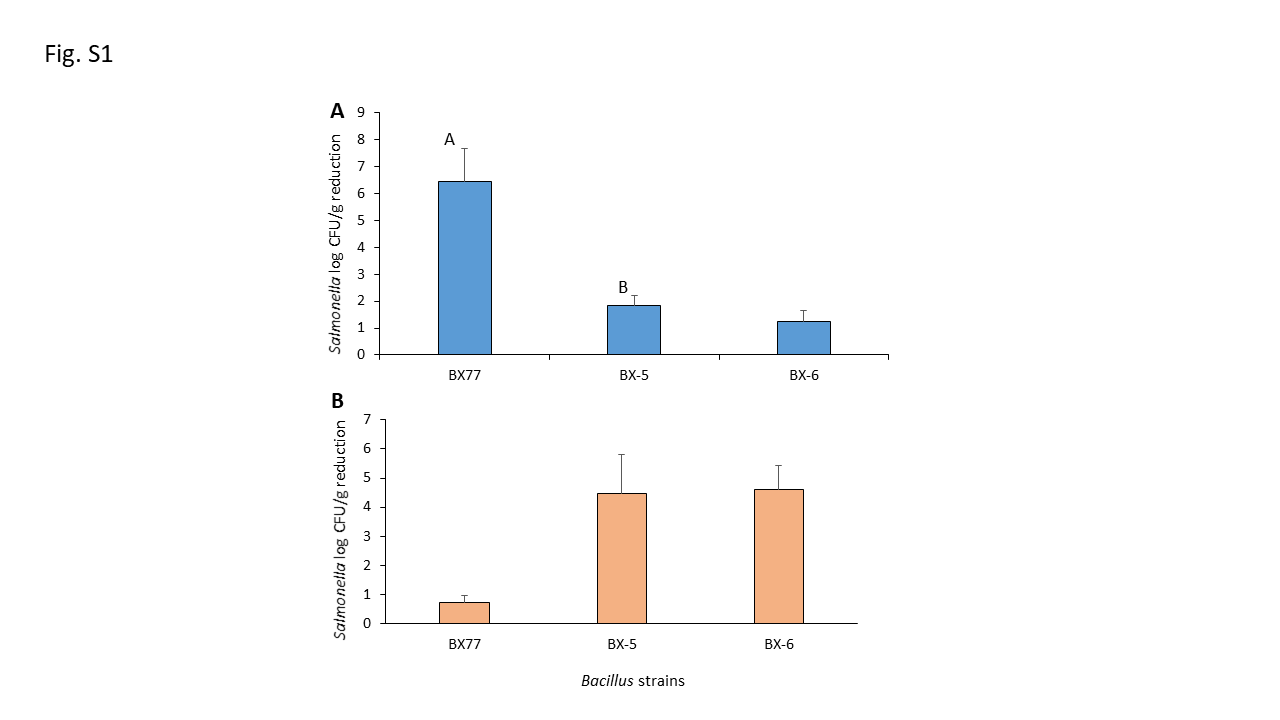

Supplement: Supplementary Figure 1 — Reduction of Salmonella in alfalfa and mung bean sprouts by various Bacillus isolates. Seeds were contaminated with Salmonella (2.0 log CFU/g) and sprouted for 4 days in the presence or absence (control) of Bacillus strains at a concentration of 7.0 log CFU/mL. (A) Sprouted alfalfa seeds. (B) Sprouted mung bean seeds. Bacillus strains BX5 and BX6 were isolated from mung bean sprouts and described previously (Chahar et al., 2023). The data represent the means and standard deviations of two independent experiments, each with three replicates. Different letters indicate significant differences (P ≤ 0.05) between the tested strains. [file Image_1.tif]

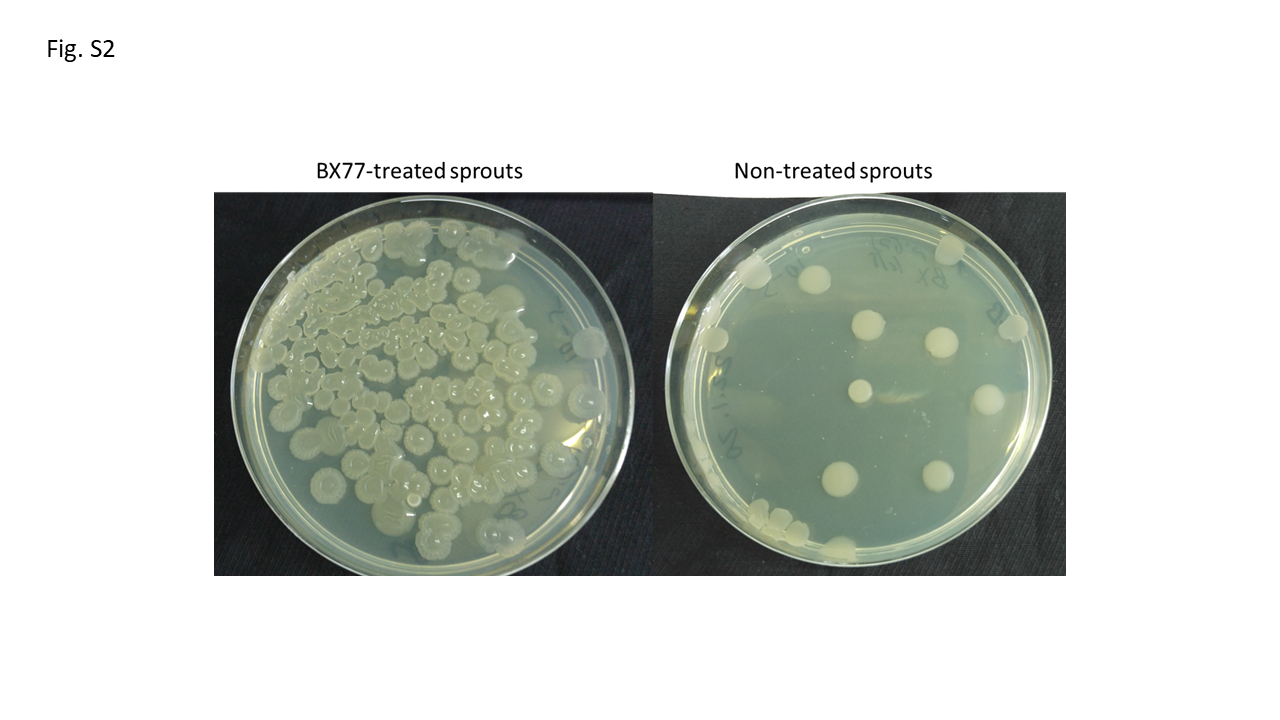

Supplement: Supplementary Figure 2 — Enumeration of Bacillus BX77 colonies on LB agar plates following extraction from treated sprouted seeds. No Bacillus BX77-like colonies were recovered from untreated sprouted-seed extract at similar dilutions. [file Image_2.tif]
